# Exhaled Nitric Oxide as Biomarker of Type 2 Diseases

**DOI:** 10.3390/cells12212518

**Published:** 2023-10-25

**Authors:** Mauro Maniscalco, Salvatore Fuschillo, Ilaria Mormile, Aikaterini Detoraki, Giovanni Sarnelli, Amato de Paulis, Giuseppe Spadaro, Elena Cantone

**Affiliations:** 1 Department of Clinical Medicine and Surgery, Federico II University, 80131 Naples, Italy; giovanni.sarnelli@unina.it; 2 Istituti Clinici Scientifici Maugeri IRCCS, Pulmonary Rehabilitation Unit of Telese Terme Institute, 82037 Telese Terme, Italy; salvatore.fuschillo@icsmaugeri.it; 3 Department of Translational Medical Sciences, Federico II University, 80131 Naples, Italy; ilaria.mormile@unina.it (I.M.); caterina.detoraki@gmail.com (A.D.); amato.depaulis@unina.it (A.d.P.); giuseppe.spadaro@unina.it (G.S.); 4 Department of Neuroscience, Reproductive and Odontostomatological Sciences-ENT Section, University of Naples Federico II, 80131 Naples, Italy; elena.cantone@unina.it

**Keywords:** allergy, asthma, biomarkers, COPD, outcome, mediators

## Abstract

Nitric oxide (NO) is a short-lived gas molecule which has been studied for its role as a signaling molecule in the vasculature and later, in a broader view, as a cellular messenger in many other biological processes such as immunity and inflammation, cell survival, apoptosis, and aging. Fractional exhaled nitric oxide (FeNO) is a convenient, easy-to-obtain, and non-invasive method for assessing active, mainly Th2-driven, airway inflammation, which is sensitive to treatment with standard anti-inflammatory therapy. Consequently, FeNO serves as a valued tool to aid the diagnosis and monitoring of several asthma phenotypes. More recently, FeNO has been evaluated in several other respiratory and/or immunological conditions, including allergic rhinitis, chronic rhinosinusitis with/without nasal polyps, atopic dermatitis, eosinophilic esophagitis, and food allergy. In this review, we aim to provide an extensive overview of the current state of knowledge about FeNO as a biomarker in type 2 inflammation, outlining past and recent data on the application of its measurement in patients affected by a broad variety of atopic/allergic disorders.

## 1. Introduction

Nitric oxide (NO) is a short-lived gas molecule that has been studied since the late 80s for its role as a signaling molecule in the vasculature and later, in a broader view, as a cellular messenger in many other biological processes such as immunity and inflammation, cell survival, apoptosis, and aging [1,2]. NO is a Janus-faced molecule involved in both physiological and pathological pathways.

NO detected by a non-invasive method in exhaled air, fractional exhaled nitric oxide (FeNO), has gained increasing importance as a biomarker of type 2 inflammation [3], providing information about disease phenotype and the response to certain treatments such as steroids or biological drugs [4,5]. FeNO levels may increase in patients with acute or chronic airway inflammation, such as type 2 bronchial asthma [6], and some respiratory infections sustained by viruses through an interferon-gamma (IFNγ)-mediated pathway [7]. On the contrary, low FeNO levels are found in patients with neutrophilic asthma, chronic obstructive pulmonary disease (COPD), and cystic fibrosis [7]. Atopic subjects tend to present higher NO levels than non-atopic subjects due to an overexpression of iNOS in their airway epithelial cells [3,7].

The arrival of biological drugs has helped to gain further insight into NO physiopathology, and, in turn, the assessment of FeNO is a useful, non-invasive, and easy-to-obtain tool in monitoring conventional and biologic therapy and predicting the outcome of a wide spectrum of type 2-mediated diseases. In this review, we aim to provide an overview of the current state of knowledge about FeNO as a biomarker in type 2 inflammation, outlining past and recent data on the application of its measurement in patients affected by a broad variety of atopic/allergic disorders including bronchial asthma and cough variant asthma (CVA), allergic rhinitis (AR), chronic rhinosinusitis (CRS) with/without nasal polyps (CRSwNP/CRSsNP), atopic dermatitis (AD), eosinophilic esophagitis (EoE), and food allergy.

## 2. Physiopathological Aspects

NO is produced by the nitric oxide synthase (NOS) [1]. There are both constitutive and inducible isoforms of this enzyme. The constitutive ones (cNOS), which include nNOS and eNOS, are expressed, respectively, by neuronal tissue and endothelium, epithelia, platelets, and skeletal muscles [8,9,10]. The inducible form (iNOS), also known as NOS2, can be expressed by a wide variety of cells in response to exogenous stimuli (e.g., bacterial lipopolysaccharide) and inflammatory mediators (e.g., cytokines). Active NOS is a tetramer composed of two NOS proteins and two calmodulin molecules [11]. An increase in the intracellular calcium concentration stabilizes the bond between calmodulin and NOS monomers, stimulating the capability of the enzyme to produce NO. For the constitutive isoforms (nNOS and eNOS), a high calcium concentration is required to bind calmodulin, while iNOS can bind calmodulin with high affinity, irrespective of calcium levels [12]. For this reason, iNOS is called “calcium-independent NOS” and it can produce NO at nanomolar levels for prolonged periods, mainly contributing to the pathophysiological effects of NO and its detectable elevation in exhaled air [11].

iNOS is physiologically expressed in bronchial epithelial cells, ensuring a basal NO production mediated by IFNγ through JAK/STAT-1 signaling. In chronic airway inflammation, including asthmatic patients’ airways, iNOS is expressed by several cells, including macrophages, neutrophils, epithelial, endothelial, and vascular smooth muscle cells, which use L-arginine and oxygen as a substrate [13]. In paranasal sinuses, iNOS acts as a constitutive form, producing a large amount of NO under the stimulation of proinflammatory cytokines (i.e., TNF-α and IL-1β), possibly enhanced by pathways linked to the microbial films [14]. However, during Th2 inflammation in upper airway diseases, the primary sources of NO are epithelial cells and macrophages [15,16]. In COPD patients, an increase in iNOS expression in the peripheral lung and small airways has been described [17,18], together with an increase in nNOS expression and activity in the airway epithelial cells and type 1 pneumocytes as a result of oxidative stress [19], suggesting that in these patients, both isoforms contribute to an increased production of NO. Finally, several skin cell populations, including keratinocytes, endothelial cells, fibroblasts, melanocytes, adipocytes, Langerhans cells, neutrophils, and macrophages, can produce NO, participating in both skin homeostasis and pathological processes. Indeed, studies on AD patients have shown that the iNOS expressed in the skin allows NO to enter the lungs via the circulation, contributing to the NO production observed in these patients [20].

NO plays a role in both physiological and pathological conditions. For instance, in the nervous system, this molecule is involved in synaptic plasticity and the signaling within neurons [21], but it also exerts an action in many neurodegenerative conditions [22]. Indeed, excessive NO and the NO-mediated posttranslational modification of cysteine thiols can induce nitrosative stress in the nervous system, contributing to the neuropathology of many neurodegenerative diseases such as Alzheimer’s disease, Parkinson’s disease, amyotrophic lateral sclerosis, Huntington’s disease, and multiple sclerosis [23]. For example, in Huntington’s disease, the S-nitrosylation of key proteins involved in the disease progression and abnormal NO signaling in the peripheral blood tissues have been observed [24]. Of note, in multiple sclerosis, some studies have found an association between mutations in the iNOS gene and the disease progression [25]. In addition, in the central nervous system of patients affected by multiple sclerosis, higher levels of iNOS RNA have been found [26]. In addition, an increase in iNOS immunoreactivity has also been shown in the active lesions compared to the normal brain [27]. Finally, in both animal models and patients with Parkinson’s disease, NO plays a complex role, participating in both neuroprotective and neurodegenerative mechanisms [23]. Researchers found that the S-nitrosylation of some proteins involved in Parkinson’s disease pathogenesis, such as parkin, causes inhibition of its ubiquitination activity, leading to the formation of Lewy bodies and disease progression [28].

In inflammatory processes, NO production increases by participating in immune defense against infectious pathogens and causing noxious effects by increasing oxidative stress [29]. A basal production of a small amount of NO (i.e., femtomolar to picomolar) produced by the bronchial epithelium is fundamental for respiratory physiology [3]. Low constitutive NO levels are also involved in bronchodilatation via NO-activated guanylyl cyclase [30]. In addition, NO enhances lung development, promotes ciliary motility, and stimulates the production of surfactant [30]. On the contrary, augmented NO levels participate in airway inflammation, free radical production, bronchial hyperreactivity, mucus hypersecretion, increased vascular permeability, reduced ciliary heartbeat, and tissue damage [3].

**Figure 1:**
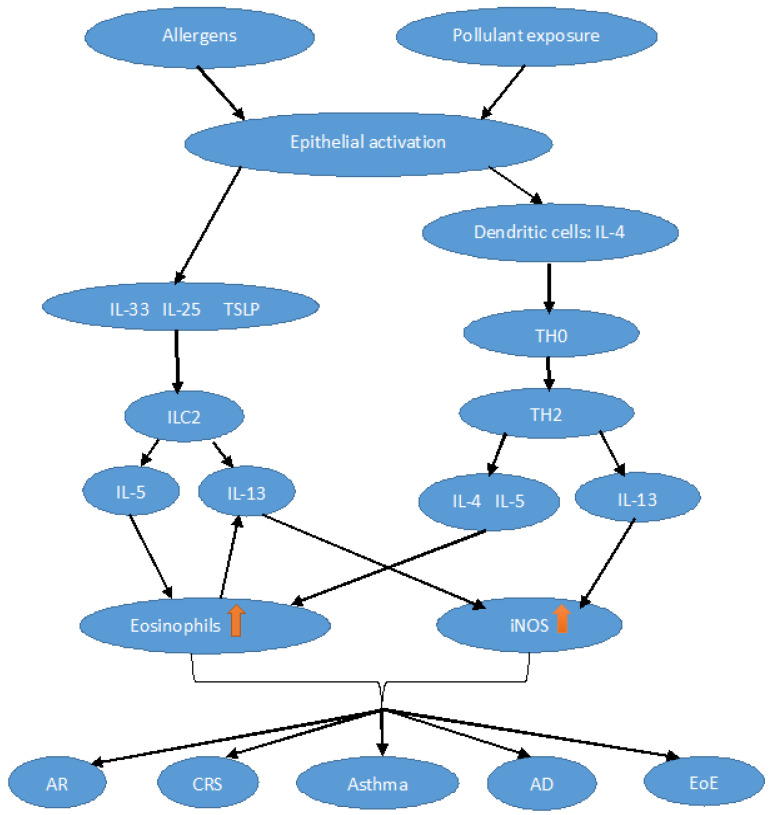
Schematic representation of the Th2 pathway. Dendritic cell activation by damaged epithelial cells secreting interleukin (IL)-4 prompts Th0 cells to differentiate into Th2 cells. Th2 cells secrete IL-5, among other cytokines, which represents the main stimulus for the production and recruitment of eosinophils (orange arrow) at the level of the target organ. Infections or exposure to pollutants induce epithelial cells to release the so-called alarmins (IL-25, IL-33, thymic stromal lymphopoietin (TSLP)) which activate innate lymphoid cells 2 (ILC2), which produce Th2 cytokines. IL-13, mainly secreted by eosinophils, activates the expression (orange arrow) of inducible nitric oxide synthase (iNOS) and increases the production of NO. AR, allergic rhinitis; CRS, chronic rhinosinusitis; EoE, eosinophilic esophagitis; AD, atopic dermatitis.

NO plays a pivotal role in Th2 inflammation (Figure 1), stimulating the survival, activity, and recruitment of eosinophils, mast cells, basophils, and lymphocytes [31]. Other evidence comes from murine models; indeed, decreased eosinophilic infiltrates, bronchial thickening, and mucus secretion, together with lower concentrations of type 2 cytokines (i.e., interleukin (IL)-4, IL-5, and -13) and chemokines (i.e., eotaxin-1), were found in asthmatic mice “knock-out” for all NOS isoforms compared to the wild type [32]. The cytokine milieu typical of Th2-mediated disease induces iNOS expression by epithelial and inflammatory cells. Indeed, iNOS expression is observed under the stimulation of various type 2 cytokines, especially IL-4 and IL-13 [33,34]. On the contrary, IL-5 induces eosinophilia but is unrelated to iNOS activation and does not contribute to NO levels [35]. Indeed, IL-4 and Il-13 induce iNOS expression mainly through the STAT 6 pathway, while IL-5 acts through different pathways, primarily activating STAT 3 [36,37].

However, eosinophil-mediated inflammation is involved in many conditions, such as upper and lower airway disease [6], allergies [38,39], gastrointestinal disorders [40] and hypereosinophilic syndromes [41], and certain cancers [42]. The role of these cells in type 2 inflammation is well known [6]. In this view, the use of FeNO as a marker of eosinophilic inflammation, particularly in some asthma phenotypes, has largely been approved [43]. Although FeNO correlates with airway eosinophilia, it is important to consider that it can be independent of eosinophils. Indeed, many authors have reported discrepancies between FeNO and sputum eosinophil count [44,45,46]. Moreover, in a study by Mac Pherson et al. [47], the authors demonstrated that although eosinophils from normal subjects failed to generate detectable levels of NO, NO2−, NO3−, or NO2Y, in the presence of physiological levels of NO2− or an exogenous NO source, eosinophils can generate NO-derived oxidants, promote oxidative modification of targets, and contribute to the inflammatory injury observed in asthmatic airway [4,5].

## 3. Exhaled NO in Type 2 Lower Airway Diseases

### 3.1. FeNO in T2 High Asthma

Based on the prevalent type of airway inflammation, asthma has been broadly categorized into two endotypes: T2-high and T2-low. T2 airway inflammation in patients with asthma, although occurring along a continuum, is characterized by a specific set of cytokines, including IL-4, IL-5, IL-13, and the so-called alarmins, IL-25 and IL-33, and thymic stromal lymphopoietin (TSLP), secreted by activated epithelial cells. The T2 inflammatory response is frequently associated with atopy (i.e., the genetic tendency to develop allergic diseases, such as allergic rhinitis, asthma, and atopic dermatitis, associated with heightened immune responses to diverse antigens/allergens, leading to CD4+ Th2 differentiation and overproduction of immunoglobulin E (IgE)) [48], eosinophilic disorders, and parasitic infections [49].

In approximately 50–70% of asthmatic patients, a T2 inflammatory response represents the pathogenetic mechanism responsible for the disease [50].

Both innate and adaptive immune responses are involved in the inflammation of type 2 asthma. Innate lymphoid cells (ILC2s) and Th2 cells are critical in orchestrating the type 2 inflammatory response. When exposed to pollutants or viral and fungal infections, ILC2s are stimulated by alarmins secreted by epithelial cells. Allergens, on the other hand, are the activators of Th2 cells. Once stimulated, ILC2 and Th2 produce type 2 cytokines, including IL-4, IL-5, and IL-13, responsible for the main alterations present in asthma. IL-4 and IL-13 cause class switching of B cells to produce IgE. IL-13 is responsible for hyperplasia of both globular and smooth cells. IL-5 is the key cytokine involved in the differentiation, maturation, and survival of eosinophils [50]. Eosinophils, mast cells, and basophils are the main effector cells of the T2 inflammation pathway [49,51].

T2-high asthma phenotypes include both early- and late-onset asthma, frequent exacerbations, atopy, eosinophilia, and increased levels of FeNO.

FeNO levels, compared to healthy controls, are elevated in T2 high asthma patients [52] and other respiratory diseases such as AR and CRSwNP [3].

In asthma, FeNO originates predominantly from the lower airways [53], and a relationship between FeNO and airway eosinophilic inflammation has been reported [54]. FeNO, eosinophils, and IgE are considered the most relevant biomarkers of the T2 inflammatory pathway.

FeNO has been used, in combination with clinical history, spirometry, and other biomarkers, to support asthma diagnosis, as a surrogate marker of airway eosinophilic inflammation, as a marker to predict response to inhaled corticosteroids, and to establish the optimal dose for asthma control. In addition, FeNO can assist in the choice of treatment with biological drugs [3,55]. In particular, the efficacy of omalizumab in reducing asthma exacerbations appears to be greater in patients with a FeNO value greater than 19.5 ppb, compared to subjects with FeNO lower than 19.5 ppb (53% vs. 16%) [56]. Similarly, in asthmatic subjects with FeNO values above 25 ppb, treatment with dupilumab, compared to placebo, reduces the risk of exacerbation by approximately 50% [57]. Similarly, tezepelumab treatment response was more pronounced in subjects with high baseline FeNO values [58].

FeNO has a 90% specificity and 85% sensitivity in the diagnosis of asthma [3]. The higher specificity versus the lower sensitivity of FeNO suggests that it has a greater role in confirming rather than excluding the diagnosis of asthma [59], especially in the early stages of the disease, when spirometric changes in reversibility have not yet appeared.

However, FeNO and eosinophilia, although related, represent two distinct biomarkers resulting from two different T2 cytokine pathways: IL-4 and IL-13, which are implied in IgE synthesis and iNOS induction and thus NO production, and IL-5, which is involved in the activation, development, and recruitment of eosinophils. The different effects on FeNO and eosinophilia of biological drugs acting on different cytokines support this view. Indeed, in asthmatic patients treated with an IL-4 receptor inhibitor (dupilumab) [33] or an IL-13 inhibitor (lebrikizumab) [34], a very significant decrease in FeNO levels can be observed. In contrast, in those treated with IL-5 inhibitors (mepolizumab), the levels of FeNO are not modified, but a significant effect on eosinophilia is appreciated [35]. In addition, this evidence confirms that IL-4 and IL-13 are the principal cytokines involved in the overexpression of iNOS. At the same time, IL-5 induces eosinophilia but is unrelated to iNOS activation and does not contribute to NO levels [36,37]. Thus, FeNO and eosinophilia are both markers of T2 airway inflammation that are often, but not always, associated [60].

Compared to other T2 biomarkers, FeNO has great potential in clinical practice because its determination is simple, non-invasive, and can be easily repeated.

The American Thoracic Society recommends FeNO to support the initial diagnosis of asthma. These guidelines define high FeNO levels in adults as >50 ppb (>35 ppb in children) and low levels as <25 ppb (<20 ppb in children) [61]. Values between 25 to 50 ppb (or 20–35 ppb for children) are considered high but should be interpreted within each patient’s clinical context and history [61].

FeNO provides prognostic information because increased FeNO levels are associated with disease activity and increased risk of exacerbations. Patients with baseline FeNO of 50 ppb or higher have a 1.54 times higher exacerbation rate than patients with FeNO of less than 25 ppb. Patients with baseline FeNO of 25 to <50 ppb hvae a 1.33 times higher exacerbation rate than patients with FeNO of less than 25 ppb [62].

The simultaneous increase in both FeNO and blood eosinophil counts could be associated with a higher prevalence of uncontrolled asthma. Indeed, Bertolini et al., in a real-world cross-sectional study, showed that elevated FeNO values were present in younger patients with lower body mass index, higher blood eosinophil counts, higher reversibility, and worse airflow obstruction and who were atopic. Furthermore, such patients often presented with persistent rhinitis and chronic rhino-sinusitis with or without nasal polyps [63].

### 3.2. FeNO in Cough Variant Asthma

CVA is a specific and atypical asthma phenotype and the most common cause of chronic cough. About 33.3% of patients with chronic cough are caused by CVA [64]. CVA is associated with airway hyperresponsiveness and chronic eosinophilic inflammation, in both central and peripheral airways, and manifests itself as a non-productive cough without wheezing or shortness of breath [65]. A positive response to the bronchial provocation test and the efficacy of bronchodilator therapy in preventing cough are the most important diagnostic criterions for CVA.

A subset of subjects with CVA (approximately 30%), especially those with higher airway responsiveness, higher sputum eosinophils, and atopy, can progress into classic asthma in the absence of appropriate treatment for asthma [66].

Small airways dysfunction (SAD) is a clinically relevant characteristic of CVA [67]. Feng-Jia et al. retrospectively analysed 150 patients with CVA and found that small airway function was significantly reduced when compared with patients without CVA [68]. In a prospective study, FeNO waa measured at exhalation flow rates of 200 mL/s (reflecting the inflammation of peripheral small airway) [69]; FeNO > 11 ppb had high diagnostic value for CVA especially in SAD patients [70]. Combining FeNO and small airway function indexes might increase the diagnostic value for differentiating CVA from typical asthma [71].

Although different inflammatory phenotypes in CVA have been described [72,73], similarly to classic asthma, the eosinophilic phenotype is frequently reported. In a study by Rybka-Fraczek et al., including 40 CVA subjects, an eosinophilic, neutrophilic, and pauci-granulocitic inflammatory pattern was observed in 32.5%, 15%, and 52.5%, respectively. Interestingly, in that study, a similar response treatment was observed irrespective of the belonging phenotype [74]. Recently, Zhan and co-workers, in 342 subjects with CVA, based on clinical and pathophysiologic data, were able to identify three distinct phenotypic clusters presenting with different clinical and pathophysiologic responses to anti-asthmatic treatment. Female predominance, late onset, normal lung function, and frequent incomplete resolution of cough after treatment identified cluster 1. Characteristic features of cluster 2 included younger subjects, nocturnal cough, atopy, high type 2 inflammation, and a high percentage of complete resolution after treatment. High body mass index, long disease duration, familial history of asthma, low lung function, and a low proportion of resolution of cough defined cluster 3. Cluster 2 presented with high FeNO levels which was one of the most important differentiating variables between clusters [75].

FeNO, being a marker of eosinophilic airway inflammation in both the central and small airways has the potential as a clinical marker in screening and diagnosing CVA in clinical practice [76,77]. FeNO discriminates patients with CVA from patients with non-CVA chronic cough such as atopic cough. FeNO levels are significantly higher in patients with CVA than those observed in patients with atopic cough [78]. It has been reported by Zhu et al. that 25.5 ppb is the optimal FeNO cut-off value in the diagnosis of CVA [76].

FeNO levels in patients with CVA are lower than those observed in patients with classic asthma, indicating greater chronic eosinophilic airway inflammation in the latter than in the former [71].

Compared with other small airway pulmonary function tests such as MMEF/MEF50, FeNO shows increased sensitivity and specificity in the diagnosis of CVA, and it is negatively correlated with both MMEF and MEF50 values. In addition, combining FeNO with MMEF/MEF50 increases the diagnostic accuracy of CVA in children [76], resulting in a rapid and accurate diagnosis, thus avoiding unnecessary treatments.

The diagnostic accuracy of FeNO in detecting CVA in chronic cough patients with AR was higher than in those with chronic cough without AR [79].

FeNO can be used as a predictor for airway eosinophilic inflammation but also as a predictor for steroid responsiveness in patients with chronic cough. The value of 34.5 ppb of FeNO has been suggested to distinguish between patients with chronic cough who respond to corticosteroid treatment and non-responders (with 85% sensitivity and about 90% specificity) [80].

### 3.3. FeNO in COPD

Airway inflammation in COPD is driven by type 1 immune response; however, in a subset of COPD patients, a T2 inflammatory response plays a significant role both in the stable state and during exacerbation of the disease [81]. Eosinophilic airway inflammation has been reported in up to 20–40% of COPD patients [82]. Blood eosinophils persistently above 2% can be found in 15–37% of patients with COPD [83], suggesting a pathogenetic role of T2 inflammatory immunity in a subset of patients.

The reasons for T2 inflammation only in a subset of COPD are poorly understood but may be due to the presence of atopy and/or asthma. An increase in Th2 and ILC2 cells has been reported in the lungs of subjects with COPD, and the epithelial cells of COPD patients produce alarmins that can activate them [84]. Pulmonary neutrophils from COPD patients show the interleukin-5 alpha receptor on their surface, which, in the presence of IL-4 and IL-5, present in the airway fluids of some COPD subjects, suggesting a further role of the T2 pathway in the pathogenesis of COPD [85]. In addition, some current and former smokers develop asthma–COPD overlap condition, associated with gene expression markers of Th2 inflammation in the airway and blood eosinophils [86]. The role of FeNO in COPD is less defined than in other chronic inflammatory diseases such as asthma. FeNO evaluation in COPD, compared with control values, has been conflicting because both increased [87] and decreased values have been reported [88]. Due to smoking habits, heterogeneity, and the severity of COPD, FeNO values in COPD patients are highly variable [89].

FeNO levels in COPD patients are higher than those observed in healthy non-smokers; however, they are not as high as those observed in untreated asthma [90].

In COPD subjects, high FeNO values, as compared to those with low values, may suggest eosinophilic inflammation and the presence of some asthmatic features [91], airway eosinophilic inflammation [92], and increased spirometric response to inhaled corticosteroids [93].

Increased FeNO levels have been reported during the exacerbated phases of COPD [94], and in clinically stable COPD outpatients, FeNO levels persistently above 20 ppb are associated with a significantly higher risk of exacerbation [95].

FeNO monitoring in COPD has a less defined role as compared to asthma. However, FeNO might also reflect in COPD the presence of airway eosinophilia and predict the response to corticosteroids. In addition, FeNO could play a role as a potential prognostic biomarker in COPD. Indeed, its level increased in patients with greater disease severity and during acute exacerbations [96] and in those at higher risk of exacerbation [95].

As compared to healthy subjects, a greater intra-individual FeNO variation in COPD patients during stable clinical conditions has been reported [97]. In addition, FeNO variability is influenced by COPD exacerbations, with FeNO increasing at the onset of exacerbation and FeNO value variability being associated with future risk of exacerbations [98].

In conclusion, in COPD, although FeNO values show greater variability, its use, particularly when combined with other T2-type inflammatory biomarkers, could improve COPD phenotypic discrimination or help to select candidates for personalized therapies, particularly in the context of biological therapy.

## 4. Exhaled NO in Type 2 Upper Airway Diseases

### 4.1. FeNO in Allergic Rhinitis

Upper and lower airways represent a continuous anatomo-physiological entity, and a high percentage (up to 38%) of patients with AR also have asthma [38,39]. Indeed, asthma and AR share a common etiopathogenesis, characterized by a T2 inflammatory substrate with the release of different biomarkers, among which NO is one of the most studied. In addition, the early and proper therapeutic management of both diseases seems to achieve mutual benefits. Likely, the measurement of NO in the upper respiratory tract provides information on the degree of eosinophilic inflammation, completing the clinical and non-invasive management of AR [99].

Patients with AR frequently express elevated levels of FeNO as an index of subclinical bronchial inflammation with potential prognostic implications [100]. Chen et al. demonstrated that higher FeNO levels and lower nasal flow are characteristics found in subjects with more severe nasal obstruction [101]. On the contrary, in another study conducted on a pediatric population, the authors did not find correlations between FeNO and nasal or asthma symptoms and rhinitis-related quality of life, suggesting that FeNO is unlikely to be a useful biomarker for the clinical severity of upper or lower airway disease [102]. Another study by Antosova et al. [103] demonstrated that FeNO levels increase with the start of the pollen season in patients with AR, even in absence of lower respiratory symptoms, and that FeNO levels were significantly reduced after H1-antihistamines administration or combined therapy with H1-antihistamines and nasal corticoids. H1-antihistamines have also been shown to directly downregulate and modulate the activity of iNOS [104]. Cardinale et al. concluded that FeNO was better correlated with serum total IgE than with allergen skin tests in children with AR and asthma, suggesting that the serum IgE can more accurately predict FeNO than allergen-specific IgE [105]. Choi et al. reported a positive correlation between FeNO and atopy biomarkers as mite-specific IgE, serum total IgE, and the blood eosinophils and their cationic protein (ECP) in children with AR and allergic asthma [106]. Scott et al. observed a significant positive correlation between FeNO and the number of allergen-positive prick tests in a cohort of asthma patients [107]. In a recent study, Saranz et al. found a significant correlation between FeNO and the number of eosinophils in peripheral blood [100].

These results taken together suggest that FeNO is rather a measure of allergy than of the severity of the disease in AR, but it could gain a value in evaluating the therapeutic effect of certain drugs in these patients.

### 4.2. FeNO in Chronic Rhino-Sinusitis

CRS is a chronic, heterogeneous, and multifactorial inflammatory disease of the upper airways, whose prevalence in the general adult population is about 10–15% [108]. In CRS, as well as in asthma, the activation of ILC-2 cells leads to the release of cytokines (Il-9, Il-4, Il-5, and IL-13) that promote a Th-2 inflammatory response [6].

CRS, like asthma, is a phenotypically heterogeneous disease. Depending on the presence or absence of nasal polyps, CRS can be classified into two types: CRS with (CRSwNP) and without nasal polyps (CRSsNP). In addition, from the histological point of view of CRSwNP, depending on the presence or absence of eosinophils infiltrating the nasal mucosa, two distinct histo-phenotypes have been reported: the eosinophilic-CRSwNP (ECRSwNP) and the non-eosinophilic-CRSwNP (NECRSwNP) [6]. The inflammatory CRS phenotypes present a substantial variability in their geographic distribution. Indeed, in Caucasian populations, CRSwNP more frequently presents with a type 2 (eosinophilic) inflammatory profile, while in Asian populations, mixed inflammatory profiles (type 1 and 3) have been reported [109].

Lower airway morbidity is highly prevalent in patients with CRSwNP [110], being present with asthma in up to 30–70% of them [111]. Accordingly, about 25% of patients with severe eosinophilic asthma have CRSwNP as a comorbidity [112].

In patients with CRSwNP, type 2 inflammation affects not only the nasal mucosa but also the bronchi and alveoli, even in the absence of asthmatic comorbidity [113]. CRSwNP with and without asthma is the most severe form of T2 inflammation in the upper airway, and CRSwNP and asthma are linked through the underlying T2 inflammatory pathway, emphasizing the continuum between the upper and lower airways [109].

Asthmatic patients affected by ECRSwNP, as compared with those with NECRSwNP, have a higher rate of nasal polyps’ recurrence, higher levels of FeNO, and more severe asthma [114].

CRSwNP is more commonly associated with severe asthma than mild-to-moderate asthma, suggesting that the presence of nasal polyps in CRS asthmatic patients may be a risk factor for asthma severity [115].

Nasal polyps in CRS patients are a major determinant of increased FeNO levels [116]. Non-asthmatic patients with CRSwNP, as compared to non-asthmatic patients with CRSsNP, show significantly higher FeNO values, while no significant difference in FeNO levels has been observed in asthmatic CRSwNP subjects as compared to non-asthmatic CRSwNP subjects [117].

Higher FeNO levels have also been reported by Kobayashi et al. in well-controlled asthmatic patients if they were affected by ECRSwNP. Therefore, in asthmatic patients, particularly those with severe disease, presenting with high FeNO levels, the co-presence of ECRSwNP should be strongly suspected [118]. The increase in FeNO found in CRSwNP might be due to the T2 eosinophilic inflammation driven by IL-5 and IL-13 cytokines released by activated ILC-2 cells. IL-5 represents the key driver of eosinophilia, while the Il-4/IL-13 inflammatory pathway is strongly associated with FeNO, which, in turn, correlates with eosinophilic inflammation in the airways. The relationship between CRSwNP and asthma is particularly relevant in the subset of patients presenting with low eosinophils count. Indeed, in a cohort of severe asthmatic patients stratified based on their blood eosinophil count, FeNO levels significantly contributed to the identification of patients with nasal polyps [119]. This is very important because the identification of key markers of polyposis will allow for better stratification of inflammatory polyp disease endotypes to objectively identify tailored medical therapies and track response to medical and surgical treatment.

FeNO is a useful marker for screening asthmatic T2-CRSwNP prior to biopsy or asthma evaluation and may assist in selecting a proper treatment. As reported by Park et al., FeNO levels and the blood eosinophil percentage were significantly higher in asthmatic patients with histological T2-CRSwNP as compared to those in patients with non-T2-CRSwNP (*p* < 0.05). A ROC curve analysis showed that a FeNO level of 27 ppb had a good ability to distinguish patients with asthmatic T2-CRSwNP (sensitivity = 90.9%; specificity = 63.9%), while the optimal cut-off values for FeNO and blood eosinophil percentage for diagnosing asthmatic T2-CRSwNP were 68 ppb and 5.6%, respectively (sensitivity = 95.5%; specificity = 86.1) [120].

In subjects with CRSwNP, high levels of FeNO have been associated with a better clinical response to dupilumab, which has been the first biological agent approved for the treatment of CRSwNP [115].

## 5. FeNO in Eosinophilic Esophagitis

Among eosinophilic gastrointestinal disorders (EGIDs), EoE is the most frequent and the most prevalent cause of chronic esophagitis, after gastroesophageal reflux disease (GERD), which is often a cause of misdiagnosis [121]. EoE is a chronic, allergen-driven eosinophilic inflammatory disease of the esophagus affecting both children and adults. Its incidence and prevalence are both rising [122].

Symptoms of EoE are related to esophageal dysfunction [123]. The gold standard diagnostic method is endoscopic biopsy with confirmed diagnosis in the presence of several eosinophils/high power field (hpf) ≥ 15 [124].

The pathogenic pathway underlying EoE is an antigen-driven Th2 immune response. Ingested allergens, especially food allergens, interacting with esophageal epithelial cells stimulate them to release alarmins (IL-25, IL-33, TSLP), thereby activating a T2-mediated immune response [125]. The relevant pathogenetic role of food allergens in sustaining the eosinophilic inflammation in EoE is suggested by the observation that dietary therapy is effective in up to 70% of patients with EoE [125].

A subset of the Th2 cell population expressing HPGDS, CRT_H_2, and IL-17RB, able to produce the highest levels of IL-5 and IL-13, has been isolated from EoE tissue [125].

Atopy is a major risk factor in EoE as most patients have a family history of bronchial asthma, AR, or atopy, and about 75% of patients with EoE have at least one coexisting type 2 inflammatory disease such as asthma, CRS, AR, and AD [126].

The study of FeNO as a non-invasive biomarker to support diagnosis and to monitor disease activity in EoE has yielded disappointing and sometimes conflicting results.

In a paediatric cohort of 134 children with suspected EoE, evaluated with endoscopic biopsy, Kaur et al. confirmed the diagnosis of EoE in 45 of them. In these patients, after adjusting for atopic diseases, FeNO levels were higher as compared to control subjects (*Z* = 3.33, *p* < 0.001) and had a statistically significant, although weak, correlation with the number of esophageal eosinophils (*r* = 0.30, *p* < 0.005) [127].

In a prospective multicenter study in non-asthmatic EoE patients, Leung and colleagues evaluated the effect of topical fluticasone treatment on FeNO levels. The results obtained, while showing a statistically significant difference in FeNO levels and esophageal eosinophil counts before and after treatment, were not able to anticipate the clinical or histological response to treatment [123].

Lanz et al., in a paediatric population composed of 55 subjects, investigated the role of FeNO in the assessment of EoE. The histopathologic diagnosis of EoE was confirmed in 18 subjects. In subjects with EoE, FeNO levels were normal in nine and elevated in the remaining nine. Based on the results obtained, the authors indicate that a normal FeNO level may be useful to exclude EoE with high specificity (>87%) and NPV (78%); however, FeNO is not suitable for the detection of subjects with EoE [128].

The ability of FeNO to reflect the level of esophageal eosinophilic inflammation was evaluated in a study by Jonhson et al. In that study, it was found that FeNO was poorly suited to distinguish between patients with esophageal eosinophils greater than or less than 15/hpf. However, the subset of patients with active disease and FeNO > 40 ppb could be identified with a high degree of certainty [129].

In another prospective study of 128 children undergoing endoscopy for suspected EoE, FeNO levels were statistically higher in active EoE than in control subjects and subjects with EoE in remission. However, the FeNO range was broad and poorly correlated with absolute eosinophil count. A panel of biomarkers including FeNO, plasma amino acids, and polyamine spermine had AUC of 0.90 and 0.87 when differentiating active EoE from controls and active EoE from EoE in remission, respectively [130]. Presently, insufficient evidence exists for recommending the routine use of FeNO measurements to predict or follow response to specific therapy in EoE. Additional studies need to be conducted to fully evaluate the potential utility of FeNO in assessing EoE disease activity.

## 6. Exhaled NO in Other Type 2 Diseases

### 6.1. Atopic Dermatitis

With a prevalence of 3% in adults and 20% in children, AD is one of the most frequent allergic conditions affecting the skin [131]. Pathogenetical mechanisms leading to this condition possibly involve environmental, genetic, and immunologic factors which cause inflammation and dysfunction of the skin barrier [132]. The presence of AD could influence FeNO levels [133,134]. Indeed, among the others, FeNO is a biomarker for the presence of a type 2 immune pattern, with increased levels of different cytokines such as IL-4 and IL-13, which are involved iNOS induction and thus NO production [135].

A recent study by Galiniak and Rachel [136] demonstrated statistically significantly higher levels of FeNO in teenage and adult populations with AD, as compared to healthy controls, with a wide distribution of FeNO in the patient group ranging from 14 to 158 ppb, as observed by other authors [137]. The authors also reported that FeNO levels correlated with the number of positive skin prick tests [136]. On the contrary, no correlation was found between FeNO levels and the duration of disease, as well as SCORing Atopic Dermatitis (SCORAD) index [137]. Moreover, no FeNO difference was found between the mild and moderate forms of AD [136]. These results aligned with evidence from a research work by Akdeniz et al. [138], in which no significant difference was found in serum NO between mild and moderate AD patients. However, in this second study, a significant difference was observed between patients with mild and severe AD, and between moderate and severe groups. Moreover, a recent and extensive review of the literature [139] on potential biomarkers in AD reported that while iNOS may be the candidate biomarker for AD diagnosis, other biomarkers may be more reliable to evaluate disease severity and treatment response. On the other hand, a positive correlation between serum nitrate level and grades of skin scores was observed in children aged 0.4–8 years [140]. In AD patients, NO production is associated with itching, while the inhibition of NOS suppresses itching [141]. However, further studies are needed to evaluate the possible role of FeNO as a marker of the severity of the disease in AD patients, as this marker may be influenced by other factors besides skin inflammation, such as the number of positive skin prick tests and the concomitance of respiratory diseases. The exact mechanism behind the increase in FeNO levels in atopic patients remains to be elucidated. However, it has been suggested that higher FeNO levels may depend on the combination of an allergic state and exposure to an appropriate allergen that leads to allergic inflammation [142]. In addition, high levels of NO may be suggestive of a mild degree of lung inflammation undetectable by standard lung function tests [143].

All of this evidence taken together indicates that currently, FeNO assessment is not a reliable tool in patients with concomitant mild or moderate AD and respiratory disease. [20,144].

### 6.2. Food Allergy

Food allergy likely affects nearly 5% of adults and 8% of children, with growing evidence of an increase in prevalence [145].

Among food allergies, a peanut allergy can be a life threatening event and accounts for approximately two-thirds of all fatal food-induced anaphylaxis [146]. Clinical peanut allergy resolves in up to 20% of children [147].

FeNO has been shown to be elevated in children with a peanut allergy who have “outgrown” their asthma [148]. In addition, FeNO may improve the ability to predict allergic reaction during a peanut challenge [149,150]. In particular, a change in FeNO during a peanut challenge was related to the severity of reaction. FeNO decreased more significantly in those who subsequently developed anaphylaxis than in those with a clinical allergy, not anaphylaxis, or who had a negative peanut challenge (tolerance) [151].

FeNO was increased also in patients with asthma and sensitization to other food allergens, with or without sensitization to airborne allergens [152]. In this study, the increase in FeNO was related to the increase in other local and systemic type 2 inflammation markers, such as serum eosinophil cationic protein (S-ECP) and periostin [152].

As a bedside test that can be used in children, it has potential for further research into mechanisms of anaphylaxis in food allergies and potentially assists in predicting an imminent anaphylactic reaction in some patients.

## 7. Conclusions

In conclusion, eosinophils-mediated inflammation is involved in many conditions including upper and lower airway disease, allergies, and gastrointestinal disorders. In this view, the use of FeNO as a marker of eosinophilic inflammation and type 2 diseases has largely been approved (Table 1). On the contrary, the role of FeNO for assessing disease severity remains to be explored in several Th2-mediated conditions. For example, endoscopy and mucosal biopsy are still the gold standard to monitor inflammation activity in EoE. For this reason, exploring the possible correlation between non-invasive markers such as FeNO and histologic findings stating the entity of inflammation would be useful in order to potentially predict tissue remodeling, organ dysfunction, and complications observed in this disorder. In addition, FeNO has shown to be effective in predicting response to certain treatments, such as some biological drugs in bronchial asthma and combination therapy with antihistamins and topic corticosteroids in AR. Therefore, evaluating its role as a marker of response to treatment in other Th2-mediated diseases such as EoE and AD could be an interesting matter of study. Future work is warranted to understand NO biology and exploit the potential of this molecule as a biomarker in type 2 inflammatory diseases and beyond.

## Figures and Tables

**Table 1:** Major cellular sources, related biomarkers, main stimulating factors, and nitric oxide synthase isoforms involved in FeNO production in various T2-driven diseases.

	FeNO	Major Cell Source	Related Biomarkers	Stimulating Factors	NOS
Asthma	↑	Epithelial cells Macrophages Eosinophils Macrophages Mononuclear cells	Sputum eosinophils Blood eosinophils Total IgE Serum periostin	IL-4 IL-13 TNF-alfa IFN-gamma TSLP	iNOS
COPD	↑ or →	Epithelial cells Pneumocytes type macrophages Vascular smooth muscle cells	Blood eosinophils Sputum eosinophils at exacerbation	TNF-alfa Reactive oxygen species	iNOS nNOS
AR	↑	Epithelial cells Macrophages Eosinophils Neutrophils	Blood eosinophils Total IgE	IL-4 IL-13	iNOS
CRSwNP	↑		Blood eosinophils Polyp eosinophils	TSLP IL-13	iNOS
EoE	↑ or →	Esophageal epithelium	Esophageal eosinophils		iNOS
AD	↑	Macrophagis Langerhans cells Keratinocytes	N° of positive skin prick tests	TNF-alfa IL-2 IL-6 IFN-gamma	iNOS
Food Allergy	↑	?	?	S-ECP Periostin	?

AR, allergic rhinitis; CRSwNP, chronic rhinosinusitis with nasal polyposis; EoE, eosinophilic esophagitis; AD, atopic dermatitis; IL, interleukin; IFN, interferon; TSLP, thymic stromal lymphopoietin; TNF, tumor necrosis factor; iNOS, inducible nitric oxide synthase; nNOS, neuronal nitric oxide synthase; S-ECP, serum eosinophil cationic protein; ↑, increased; →, normal; ?, unknown.

## Data Availability

Not applicable.
